# Association of TGFβ1, TNFα, CCR2 and CCR5 gene polymorphisms in type-2 diabetes and renal insufficiency among Asian Indians

**DOI:** 10.1186/1471-2350-8-20

**Published:** 2007-04-12

**Authors:** Pushplata Prasad, Arun K Tiwari, KM Prasanna Kumar, AC Ammini, Arvind Gupta, Rajeev Gupta, BK Thelma

**Affiliations:** 1 Department of Genetics, University of Delhi South Campus, New Delhi, India; 2 Department of Endocrinology and Metabolism, M.S. Ramiah Medical College, Bangalore, India; 3 Department of Endocrinology, All India Institute of Medical Sciences, New Delhi, India; 4 Jaipur Diabetes and Research Centre, Jaipur, India; 5 Monilek Hospital and Research Centre, Jaipur, India

## Abstract

**Background:**

Cytokines play an important role in the development of diabetic chronic renal insufficiency (CRI). Transforming growth factor β1 (TGF β1) induces renal hypertrophy and fibrosis, and cytokines like tumor necrosis factor-alpha (TNFα), chemoattractant protein-1 (MCP-1), and regulated upon activation and normal T cell expressed and secreted (RANTES) mediate macrophage infiltration into kidney. Over expression of these chemokines leads to glomerulosclerosis and interstitial fibrosis. The effect of MCP-1 and RANTES on kidney is conferred by their receptors i.e., chemokine receptor (CCR)-2 and CCR-5 respectively. We tested association of nine single nucleotide polymorphisms (SNPs) from TGFβ1, TNFα, CCR2 and CCR5 genes among individuals with type-2 diabetes with and without renal insufficiency.

**Methods:**

Type-2 diabetes subjects with chronic renal insufficiency (serum creatinine ≥ 3.0 mg/dl) constituted the cases, and matched individuals with diabetes of duration ≥ 10 years and normoalbuminuria were evaluated as controls from four centres in India. Allelic and genotypic contributions of nine SNPs from TGFβ1, TNFα, CCR2 and CCR5 genes to diabetic CRI were tested by computing odds ratio (OR) and 95% confidence intervals (CI). Sub-analysis of CRI cases diabetic retinopathy status as dependent variable and SNP genotypes as independent variable in a univariate logistic regression was also performed.

**Results:**

SNPs Tyr81His and Thr263Ile in TGF β1 gene were monomorphic, and Arg25Pro in TGF β1 gene and Δ32 polymorphism in CCR5 gene were minor variants (minor allele frequency <0.05) and therefore were not considered for case-control analysis. A significant allelic association of 59029G>A SNP of CCR5 gene has been observed and the allele 59029A seems to confer predisposition to development of diabetic CRI (OR 1.39; CI 1.04–1.84). In CRI subjects a compound group of genotypes "GA and AA" of SNP G>A -800 was found to confer predisposition for proliferative retinopathy (OR 3.03; CI 1.08–8.50, p = 0.035).

**Conclusion:**

Of the various cytokine gene polymorphisms tested, allele 59029A of CCR5 gene is significantly associated with diabetic renal insufficiency among Asian Indians. Result obtained for 59029G>A SNP of CCR5 gene is in conformity with reports from a Japanese population but due to sub-optimal power of the sample, replication in larger sample set is warranted.

## Background

Lifestyle and overeating with consequent obesity are major triggering factors for type-2 diabetes epidemic but important underlying pathogenic elements appear to be genetic factors. Positive family history confers a 2.4 fold increased risk and 15–25% of first degree relatives of patients with type-2 diabetes develop impaired glucose tolerance. The lifetime risk for type-2 diabetes is 38% if one parent has diabetes and 60% if both the parents are affected [1]. Multiple susceptibility genes have been reported in pathogenesis of type-2 diabetes as well as diabetic complications such as nephropathy. Clinical diabetic nephropathy results from glomerular, tubular, interstitial and vascular lesions. Genesis of diabetic kidney disease involves both hemodynamic and metabolic pathways and a subset of diabetic patients who develop nephropathy may have a genetic susceptibility to develop renal injury in response to abnormal physiological milieu that is associated with diabetes. There is interplay of metabolic, biochemical and hemodynamic abnormalities that contribute to development of diabetic renal disease and it has been proposed that renin-angiotensin-aldosterone system (RAAS), nitric oxide, and transforming growth factor (TGF)-β1 pathways are important [2].

As a part of an extensive analysis of genetic susceptibility to diabetic renal disease, we had earlier reported the role of RAAS gene polymorphisms in diabetic CRI. A highly significant association of Met235Thr, and a weaker association of T>C (-344) and G>A (-1903) SNPs with CRI, independent of hypertension, was observed there in [3]. It suggested that the effect of RAAS may not be mediated through modulation of hypertension as a crucial mechanism for development of CRI but it may operate by stimulation of chemokines like transforming growth factor β1 (TGFβ1), tumor necrosis factor α (TNFα) and interleukin 1 (IL1). Hyperglycemia and systemic hypertension leads to glomerular hypertension as a result of ineffective preglomerular resistances, which are possibly genetically determined [4,5]. Glomerular hypertension in early stages of nephropathy leads to changes in endothelial and mesangial cells. The resultant increased expression of glucose transporter-1 (GLUT-1) leads to increased production of TGFβ1 and formation of advanced glycation end-products (AGE) and its receptor [6,7]. This promotes deposition of collagens I, III and fibronectin. Activation of mitochondrial protein kinase-C (PKC) also leads to production of TGFβ1, superoxide and peroxynitrite and decreases activity of nitric oxide. This decreased activity increases angiotensin II and angiotensinogen II type-1 receptor which loop back upstream in the cascade increasing glomerular hypertension and expression of TGFβ1 and GLUT-1 thereby continuing the vicious cycle of glomerular injury [2]. In addition, angiotensin II also activates PKC and MAPK pathways, which are abundantly implicated in activation of TGFβ1, TNFα and IL1, in various cells [8-11]. Over expression of TNFα and IL-1 stimulates the expression of chemokines like monocyte chemoattractant protein1 (MCP1) and "Regulated upon activation and normal T cell expressed and secreted" (RANTES) in human mesangial cells [12-14]. Up regulation of MCP1 and RANTES triggers recruitment of monocytes and macrophage infiltration in glomerulus of diabetic rats [15] and DN subjects [16]. Chemokine receptors, CCR2 and CCR5, are the major receptors for MCP1 and RANTES respectively and are expressed on the surface of monocytes. Wada et al [17] have reported that CCR5 positive cells were detected in both the glomeruli and the interstitium of human crescentic glomerulonephritis (GN) and therefore, it is speculated that CCR2 and CCR5 might be involved in the recruitment of macrophages in human DN. CCR2 and CCR5 mediated monocyte recruitment and differentiation to macrophage in the glomerulus and interstitium has been speculated to play a role in development of glomerulosclerosis and fibrosis in progressive diabetic kidney disease [18]. Considering such importance of inflammatory genes in development and progression of diabetic renal disease, we tested association of nine SNPs from TGFβ1, TNFα, CCR2, and CCR5 genes with diabetic chronic renal insufficiency (CRI) using a case-control design.

## Methods

### Subjects

Ethical committee clearance for the study was obtained from the participating medical institutions and universities. In this case-control study, consecutive subjects suffering from type-2 diabetes with CRI (cases, CRI, n = 196) and diabetics without any evidence of diabetic kidney disease (controls, DM, n = 225) were recruited (after obtaining written informed consent from the study subjects) from the outpatient departments of the four participating medical institutions situated across the country. These included MS Ramiah Medical College (Bangalore), All India Institute of Medical Sciences (New Delhi), Jaipur Diabetes and Research Centre (Jaipur), and Monilek Hospital and Research Centre (Jaipur). The research carried out on study subjects was in compliance with the Helsinki Declaration [19]. Diabetes was diagnosed on the basis of World Health Organization guidelines. Demographic details and clinical profile of the study population have been described earlier ([3]; See additional file 1). In brief, inclusion criteria for the CRI group (cases) were subjects with type-2 diabetes of ≥ 2 years, serum creatinine ≥ 3 mg/dl, urinary albumin excretion rate (AER) > 200 mg/l and presence of diabetic retinopathy. Patients with drug induced nephrotoxic damage or secondary causes of albuminuria such as obstructive renal disease, renal stone disease and acute urinary tract infection were excluded. Normoalbuminuric (AER<20 mg/l) individuals with type-2 diabetes of ≥ 10 years duration (average 17.07 ± 6.69 years) were recruited as control subjects.

### Genetic analysis

DNA from venous blood was isolated using phenol-chloroform method and used for genetic analysis. A total of nine SNPs namely -800 G>A, -509 C>T, Arg25Pro, Tyr81His and Thr263Ile in TGFβ1gene, G>A (-308) in TNFα, Val64Ile in CCR2 gene, and CCR5Δ32 and 59029 G>A in *CCR5 *gene, were genotyped using the polymerase chain reaction-restriction fragment length polymorphism (PCR-RFLP) approach. Primers for all the SNPs were designed using Primer 3 software. Details including location of SNPs in the respective genes, primer sequences, PCR conditions and restriction enzyme with product sizes are presented in Table 1. The digested PCR products were resolved on 2–3% agarose gels stained with ethidium bromide.

**Table 1:** SNPs in TGFβ1, TNFα, CCR2 and CCR5 genes, their location, primer sequences, PCR conditions and restriction enzyme with product sizes.

**Polymorphism**	**Primer sequence**	**Product size (bp)**	**Annealing temp./restriction enzyme/allele sizes**
**TGFβ1** **G>A (-800)** **Promoter**	F: 5' GGCAGTTGGCGAGAACAGT-3' R: 5' ACCCAGAACGGAAGGAGAGT3'	600	57°C/Tai I/ G = 199, 401 A = 600
**TGFβ1** **C>T (-509)** **Promoter**	F: 5'-GGCAGTTGGCGAGAACAGT-3' R: 5'-ACCCAGAACGGAAGGAGAGT-3'	600	57°C/Eco 8I1/ C = 489,111 T = 600
**TGFβ1** **Arg25Pro** **Exon 1**	F: 5'TTC CCT CGA GGC CCT CCT A3' R: 5' CAC AGC AGC GGT AGC AGC TG3'	294	61°C/Sac II/ G = 202,67,25 C = 269,25
**TGFβ1** **Tyr81His** **Exon 2**	F: 5' CCAGATCCTGTCCAAGCTG3' R: 5' TGGGTTTCCACCATTAGCAC3'	198	57°C/Rsa I/ T = 89,109 C = 198
**TGFβ1** **Thr263Ile** **Exon 5**	F: 5' CACCAAAGCAGGGTTCACTA3' R: 5' ATCCAGGCTACAAGGCTCA3'	238	58°C/Fok I/ C = 238 T = 84, 154
**TNFα** **G>A -308** **Promoter**	F:5'AGGCAATAGGTTTTGAGGGcCAT3' R:5'GGGACACACAAGCATCAAGGATAC3'	146	37°C/Nco I/ G = 146 A = 122, 24
**CCR2** **Val64Ile** **Exon 1**	F: 5'TTG TGG GCA ACA TGA TGG3' R: 5'GCA TTC CCA AAG ACC CAC TC3'	163	57°C/BsaBI/ T = 163 C = 145,18
**CCR5** **G>A (59029)** **Promoter**	F: 5' CAG TCA ACC TGG GCA AAG CC3' R: 5' AGC TTT GGT CCT GAG AGT CC3'	453	57°C/Bst 1286I/ G = 408, 45 A = 453
**CCR5** **Δ32** **Promoter**	F:5' GAAGTTCCTCATTACACCTGCAGCTCTC3' R:5' CTTCTTCTCATTTCGACACCGAAGCAG AG3'	174/142	55°C/ 1 = 174 2 = 142

### Statistical analysis

Difference in continuous and nominal clinical variables among DM (controls) and diabetic CRI (cases) groups was compared using t-test and χ^2 ^tests respectively. Hardy Weinberg equilibrium (HWE) was tested for each SNP using the data obtained by genotyping about 200 healthy, non-diabetic individuals from different states of India [unpublished data]. Pair wise linkage disequilibrium between multiple SNPs of a gene was calculated using EMLD Software [20]. Allelic and genotypic associations at each of the SNPs were tested using Pearson's χ^2 ^test and calculation of odds ratio (OR). Power of sample size was calculated using PAWE software [21,22]. Pair wise interactions between SNPs were assessed using logistic regression analysis. Cumulative effect of different clinical variables and SNPs was also tested by logistic and linear regression analyses using SPSS. P values < 0.05 were considered significant.

## Results

Clinical details of the subjects included in the study are reported and discussed somewhere else ([3]; See additional file 1). In brief, No significant difference was observed in body-mass index, total serum cholesterol and triglyceride levels between the DM and CRI groups (p > 0.05). However, serum creatinine and blood pressure and proportion of hypertensive individuals and those with diabetic retinopathy were significantly greater (p < 0.05) among diabetic CRI subjects compared to DM controls.

### Genetic analysis

#### TGFβ1

Of the five SNPs selected for genotyping in this gene, two SNPs namely, Tyr81His and Thr263Ile were monomorphic and Arg25Pro was a minor variant (allele frequency of variant allele= 0.01) and therefore not analysed further. Since the r^2 ^value between G>A -800 and C>T -509 SNPs (D' = 0.99, r^2 ^= 0.05) was not significant both of these were genotyped in the case-control population. Neither of these two SNPs showed ether allelic or genotypic association (Table 2).

**Table 2:** Allele and genotype frequencies (F) of SNPs and their association status with chronic renal insufficiency

**SNPs**	**Allele frequency**		**Genotype frequency**		**Association**	
	**DM** F (* n)	**CRI** F (* n)	**DM** F (** n)	**CRI** F (** n)	**Allele** **(df = 1)**	**Genotype** **(df = 2)**
TGFβ1 G>A (-800)	G = 0.93 (418) A = 0.07 (32)	G = 0.91 (356) A = 0.09 (72)	GG = 0.87 (196) GA = 0.12 (27) AA = 0.01 (3)	GG = 0.84(165) GA = 0.15 (29) AA = 0.01 (2)	χ^2 ^= 0.60; P = 0.44	χ^2 ^= 1.16; P = 0.56
TGFβ1 C>T (-509)	C = 0.875 (388) T = 0.125 (112)	C = 0.84 (330) T = 0.16 (124)	CC = 0.77 (171) CT = 0.21 (47) TT = 0.02 (4)	CC = 0.72 (141) CT = 0.24 (47) TT = 0.04 (8)	χ^2 ^= 1.24; P = 0.27	χ^2 ^= 1.23; P = 0.54
TNFα G>A (-308) Promoter	G = 0.93 (416) A = 0.07 (32)	G = 0.96 (376) A = 0.04 (16)	GG = 0.87 (195) GA = 0.12 (27) AA = 0.01 (2)	GG = 0.91 (178) GA = 0.08 (16) AA = 0.01 (2)	χ^2 ^= 2.31; P = 0.13	χ^2 ^= 2.43; P = 0.12
CCR2 C>T Val64Ile	C = 0.91 (410) T = 0.09 (40)	C = 0.94 (368) T = 0.06 (24)	CC = 0.84 (189) CT = 0.15 (34) TT = 0.01 (2)	CC = 0.90 (176) CT = 0.09 (18) TT = 0.01 (2)	χ^2 ^= 2.73; P = 0.10	χ^2 ^= 2.87; P = 0.24
CCR 5G>A (59029)	G = 0.46 (208) A = 0.54 (242)	G = 0.38 (148) A = 0.62 (244)	GG = 0.21 (47) GA = 0.49 (111) AA = 0.30 (67)	GG = 0.14 (27) GA = 0.48 (94) AA = 0.38 (75)	χ^2 ^= 5.13; P = 0.02	χ^2 ^= 5.22; P = 0.07

* Number of respective alleles, and ** genotypes in the study population.

#### TNFα

No allelic or genotypic association of G>A (-308) promoter SNP of the gene with CRI was observed in this study (Table 2).

#### CCR2

No allelic or genotypic association of Val64Ile with CRI was observed in this study (Table 2).

#### CCR5

A significant association of allele 'A' of 59029 G>A SNP with CRI was observed (OR 1.39, CI 1.04–1.84, p = 0.02). CCR5Δ32 polymorphism was only a minor variant and therefore, not included for case-control association.

In a univariate logistic regression analysis, keeping disease status (DM or CRI) as dependent variable and all the genotypes as independent variables, we observed a significant association of the genotype GA (OR 1.95, CI 1.07–3.55, p = 0.028) of 59029 G>A SNP with CRI. In a multiple logistic regression analysis keeping disease status (DM or CRI) as dependent variable and all the genotypes and crucial clinical parameters as independent variables no association of any of the polymorphisms or clinical parameters with CRI was observed. Pair wise interactions tested between different SNPs using logistic regression were not found to be significantly associated with diabetic kidney disease.

An additional, sub-analysis of diabetic CRI category using retinopathy status (proliferative vs. non-proliferative) as dependent variable and genotypes (all the polymorphisms analysed in this study) as independent variables was performed using univariate logistic regression (Table 3). A compound group of genotypes 'GA and AA' of SNP G>A -800 was found to confer predisposition in only those diabetic CRI patients with proliferative retinopathy (OR 3.03, CI 1.08–8.50, p = 0.035).

**Table 3:** A sub-analysis of case (CRI) category using proliferative retinopathy as an outcome variable

**SNP**	**P**	**Exp(B)**	**95% C.I. for Exp(B)**	
			**Lower**	**Upper**
TGF β1 G>A (-800)	0.031	0.336	0.124	0.908
TGF β1 C>T (-509)	0.21	0.743	0.466	1.183
CCR2 C>T Val64Ile	0.797	0.879	0.330	2.345
TNF α G>A (-308)	0.58	0.70	0.20	2.48
CCR5 G>A (-59029)	0.988	1.044	0.627	1.606

## Discussion

Chronic renal insufficiency due to diabetes is the most frequent cause of death due to end stage renal disease and thus a major health concern world over [23]. Contemporary evidences both from *in vitro *and *in vivo *studies in experimental animals and renal biopsy specimens from patients with progressive diabetic renal disease suggest that cytokines play crucial role in development and progression of diabetic kidney disease [24-26]. Moreover, results from our previous report [3] suggested that the effect of RAAS may not be mediated through modulation of hypertension as a crucial mechanism for development of CRI but it may operate by stimulation of inflammatory genes. Therefore, in this case-control study genetic contribution of nine SNPs from TGFβ1, TNFα, CCR2 and CCR5 genes for CRI among individuals with type2 diabetes have been analysed. We observed a significant allelic association of allele "A" of SNP 59029 G>A in chemokine receptor-5 gene with diabetic CRI.

Neither of the two promoter SNPs (G>A -800 and C>T -509) of TGFβ1 gene showed association with CRI in this study. These SNPs have not been tested for association with diabetic nephropathy in any other population and thus no comparison is possible. However, a study reported a significant association of C>T-509 (p = 0.02) SNPs in TGFβ1 gene polymorphisms with chronic kidney failure in European population [27]. Another study, which investigated association of two polymorphisms inTGFβ1 gene, reported a significant association of Arg25Pro inTGFβ1 gene with end-stage renal disease [28]. However, in our population since SNP Arg25Pro was found to be a minor variant (with frequency of variant allele = 0.01, unpublished data), it was non-informative. Lack of association of CCR2 Val64Ile SNP in our study is similar to other reports from Japanese population [14]. As for association of CCR5 gene polymorphisms with CRI, we observed a strong trend of association of 59029 G>A SNP. This is in conformity with two other available reports of association of this marker with DN among Japanese population [14,29]. Though the allele 'A' is predominant in both control (DM) and case (CRI) groups, in our sample set, the allele frequency is significantly higher in CRI group (P = 0.02) and seems to be predisposing based on OR estimates (OR: 1.39; CI: 1.04–1.84). An enhanced expression of CCR5 by peripheral blood mononuclear cells has been seen in individuals with the CCR5 59029A-genotype [30,31], thereby suggesting that the genotype could regulate CCR5 gene expression. This further corroborates our observation of association of allele 'A' with the disease. Considering the important role of CCR5 gene in macrophage infiltration into glomerulus and interstitium, our observation of association of promoter SNP in seems exciting but in view of the sub-optimal power of the sample analysed (G = 29%), a replication study seems warranted.

Following analysis on the two sub groups of CRI patients, one with and the other without proliferative retinopathy (PR), a compound group of genotypes 'GA and AA' of SNP G>A -800 in TGFβ1 gene was found to confer predisposition (P = 0.035; OR: 3.028; CI: 1.079–8.50) only in those CRI patients with proliferative retinopathy (Table 3). TGFβ1 gene has been known to influence almost every pathway implicated in development of diabetic CRI [32]. Therefore, considering that TGFβ1 plays a very crucial role in diabetic CRI progression and development, our finding seems promising. However, due to relatively small sample size after sub-grouping in this analysis, we would be cautious in interpreting our results.

## Conclusion

In conclusion, our results suggest that 59029 G>A SNP in CCR5 gene may play a role in CRI susceptibility in the Asian Indian population.

## Competing interests

The author(s) declare that they have no competing interests.

## Authors' contributions

All authors have read and approved the final Ms. PP was involved in the study design, carried out molecular genetics and statistical analyses, compiled the data, wrote the Ms.; AKT was involved in molecular genetic analysis; KMPK, ACA, AG, and RG were the principal clinical investigators involved in study design, defining exclusion and inclusion criteria of study subjects and were mainly responsible for identification of study subjects from their respective clinical centres; BKT was the principal geneticist and coordinator of the project, involved in conceptualization of the project, study design, oversee complete genetic analyses in the laboratory, critical inputs and finalization of the manuscript.

## Pre-publication history

The pre-publication history for this paper can be accessed here:



## Supplementary Material

Additional File 1 Clinical characteristics of the study population. The data provided represent the demographic and clinical characteristics of the study population. Click here for file
