# Supplementary material for: Association of TGFβ1, TNFα, CCR2 and CCR5 gene polymorphisms in type-2 diabetes and renal insufficiency among Asian Indians
Source: BMC Med Genet. 2007 Apr 12;8:20. doi: 10.1186/1471-2350-8-20 (PMC1853079; doi:10.1186/1471-2350-8-20)
Supplement: Additional File 1 — Clinical characteristics of the study population. The data provided represent the demographic and clinical characteristics of the study population. [file 1471-2350-8-20-S1.doc]

Supplementary Table: Clinical characteristics of the study population

| Characteristics | DM^a^  (n=225) | CRI^b^  (n=196) | P |
| --- | --- | --- | --- |
| Gender (M/F) | 76/149 | 65/131 | 0.89^d^ |
| Age (years) | 60.6±11.5 | 57±12.8 | 0.10^c^ |
| Duration of Diabetes (years) | 17.07±6.69 | 10.4± 7.7 | <0.05^c^ |
| Hb A_1c_ (%) | 7.3±1.0 | 7.5±1.1 | 0.949 ^c^ |
| Systolic pressure (mm Hg) * | 140, 84 | 150, 100 | 0.002^e^ |
| Diastolic pressure (mm Hg) * | 84, 24 | 90, 40 | 0.025^e^ |
| Serum creatinine (μmol/l) * | 84, 24 | 177, 124 | <<0.05^e^ |
| UAER (mg/l) * | 10, 16 | 864, 720 | <<0.05^e^ |
| GFR (mls/min/1.73 m^2^) | 81.44±21.85 | 27.83±24 | <<0.05^c^ |
| Serum triglyceride (mmol/l) | 1.7±0.67 | 1.8±1.4 | 0.307^c^ |
| Serum cholesterol (mmol/l) | 4.8±0.97 | 4.9±0.92 | 0.852^c^ |
| Retinopathy (%)  Non proliferative (%)  Proliferative (%) | 24  15  8.6 | 88  35  53 | <<0.05^d^ |
| Cardiovascular events (%) | 3 | 8.3 |  |

**Data presented as mean ± SD (*median and range). ^a^type 2 diabetes subjects without nephropathy (DM); ^b^with diabetic renal insufficiency (CRI); ^c^Student’s t test; ^d^Pearson’s χ^2^ test; ^e^Mann-Whitney U test.**
